# Body Mass Index Combined With Possible Sarcopenia Status Is Better Than BMI or Possible Sarcopenia Status Alone for Predicting All-Cause Mortality Among Asian Community-Dwelling Older Adults

**DOI:** 10.3389/fnut.2022.881121

**Published:** 2022-06-30

**Authors:** Chalobol Chalermsri, Wichai Aekplakorn, Varalak Srinonprasert

**Affiliations:** ^1^Division of Geriatric Medicine, Department of Preventive and Social Medicine, Faculty of Medicine Siriraj Hospital, Mahidol University, Bangkok, Thailand; ^2^Department of Women's and Children's Health, Uppsala University, Uppsala, Sweden; ^3^Department of Community Medicine, Faculty of Medicine Ramathibodi Hospital, Mahidol University, Bangkok, Thailand; ^4^Division of Geriatric Medicine, Department of Medicine, Faculty of Medicine Siriraj Hospital, Mahidol University, Bangkok, Thailand; ^5^Siriraj Health Policy Unit, Faculty of Medicine Siriraj Hospital, Mahidol University, Bangkok, Thailand

**Keywords:** body mass index, BMI, possible sarcopenia status, all-cause mortality, Asian community-dwelling older adults

## Abstract

**Background:**

Body mass index (BMI) and sarcopenia are common indicators of nutritional status. Possible sarcopenia, defined as low muscle strength or performance, was recently introduced by the Asian Working Group for Sarcopenia (AWGS) in 2019. We investigated for association between all-cause mortality and BMI combined with possible sarcopenia severity in Asian older adults.

**Methods:**

This study included a subpopulation (8,195 participants aged ≥60 years; male gender: 49.4%; mean age: 69.2 ± 6.8 years) from the Fourth Thai National Health Examination Survey (NHES-IV). BMI was classified using Asia-Pacific cut-offs. Possible sarcopenia was defined using quadriceps strength based on AWGS 2019 criteria, and possible sarcopenia severity was determined using study population quartile cut-offs. All-cause mortality data was derived from the national vital registry in 2020.

**Results:**

The prevalence of underweight status and possible sarcopenia was 11.8 and 38.9%, respectively. Multivariate analysis showed underweight individuals with severe possible sarcopenia to be at highest risk for increased mortality [adjusted hazard ratio (aHR): 3.98, 95% confidence interval (CI): 2.89–5.48], and higher risk was found in men compared to women (aHR: 5.35, 95% CI: 1.19–8.97). Obese status without possible sarcopenia was an independent protective factor (aHR: 0.61, 95% CI: 0.38–0.97).

**Conclusion:**

BMI combined with possible sarcopenia severity is a better predictor of mortality risk than either parameter alone.

## Introduction

Malnutrition is a common and important public health problem among older adults due to its strong association with morbidity and mortality (1). Body mass index (BMI) is an anthropometric parameter that is routinely used to assess nutritional status (2). Although BMI is strongly correlated with health outcomes, direct association between BMI and mortality among older adults is still being investigated and debated. Many studies have reported a reverse J curve or U curve association between BMI and mortality; however, other studies that examined these associations did not find a similar relationship between BMI and mortality (3–5). Furthermore, the accuracy of BMI measurement in older adults remains problematic. BMI is calculated from body weight and height. A decrease in height due to aging can increase the BMI value without any change in body weight (6). As a result, no consensus has yet been reached regarding the optimal cut-off point for optimal BMI in older adults.

Apart from BMI—sarcopenia, which is defined as loss of muscle mass plus low muscle strength and/or low physical performance, is also strongly associated with malnutrition and negative health consequences (7). Although sarcopenia is a good predictor of nutritional status, diagnosis requires physical performance measurement, and muscle mass measurement. Dual energy X-ray absorptiometry (DEXA) and bioelectrical impedance analysis (BIA) are the commonly used tools for measuring muscle mass in a research setting; however, these tools are not widely available in community setting, especially in developing countries.

Recently, the Asian Working Group for Sarcopenia (AWGS) introduced the term “possible sarcopenia,” which is defined as low muscle strength or reduced physical performance (8). Possible sarcopenia allows for an easier and earlier diagnosis of sarcopenia, particularly in community setting. Limited evidence currently exists specific to the association between possible sarcopenia and mortality in Asian older adults. Therefore, the aim of this study was to determine the optimal BMI in Asian older adults that associates with the lowest all-cause mortality, and to investigate for association between all-cause mortality and BMI combined with various degrees of possible sarcopenia in Asian community dwelling older adults.

## Materials and Methods

### Study Design

**Figure 1:**
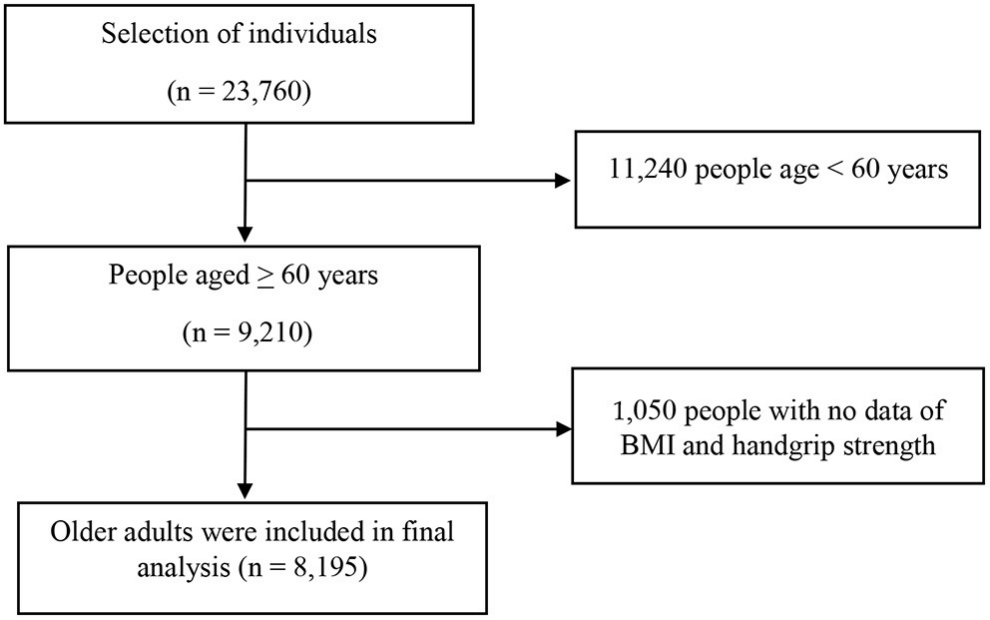
The flowchart of participants' selection process.

This retrospective cohort study used data from the Fourth Thai National Health Examination Survey (NHES-IV). The NHES-IV was a national health examination survey of Thai population that was conducted during August 2008 to March 2009. In the survey, multistage cluster sampling was employed. The sampling technique was described elsewhere (9). A total of 20,450 individuals were enrolled in the NHES-IV. The response rate was 85.5% for men, and 95.4% for women. The inclusion criteria for the present study were age ≥60 years, having BMI data and handgrip strength data. A total of 1,015 participants aged ≥60 years were excluded due to a lack of BMI and/or handgrip strength data. A total sample size of 8,195 was included for the final analyses (Figure 1).

### Data Collection and Measurement

Data were obtained for the NHES-IV study *via* a semi-structured face-to-face interview between the respondent and trained personnel. Body weight and height were both measured by standardized procedures, and BMI was calculated as weight in kilograms divided by height in meters squared. BMI was classified using the following Asia-Pacific cut-off values: BMI <18.5 kg/m^2^ as underweight, 18.5–22.9 kg/m^2^ as normal weight, 23.0–24.9 kg/m^2^ as overweight, and ≥25.0 kg/m^2^ as obese (10). In the present study, to examine the optimal BMI for achieving the lowest all-cause mortality, the BMI range in our study population (14–36 kg/m^2^) was classified into subgroups for every 2 kg/m^2^ (e.g., 14–15.9, 16–17.9 kg/m^2^, etc.). Hand grip strength was measured using a hydraulic hand dynamometer (Takei Scientific Instrument; Product No. T.K.K.5401). The interface reports grip strength in kilograms to one decimal point. All participants were seated with the elbow flexed at 90°, the, forearm in neutral position and the wrist between 0 and 30°. Each participant was asked to have two assessments per arm at 1-min intervals. The maximum handgrip strength was used for analysis. Possible sarcopenia was defined by low muscle strength using handgrip strength <28 kg for men, and <18 kg for women according to the AWGS 2019 guideline (8). We also set forth to examine the association between severity of possible sarcopenia and all-cause mortality. Therefore, handgrip strength was categorized using quartile cut-offs. The lowest quartile represented the study subjects with the lowest handgrip strength. Handgrip strength were categorized into quartile. The cut-off values of handgrip strength in each quartile among male older people were <24.6, 24.7–29.2, 29.3–33.8, and >33.9 kg. The cut-off values of handgrip strength in each quartile for female older people were <16.6, 16.7–19.7, 19.8–22.8, >22.9 kg. The analysis was performed using the highest quartile as the reference group (defined as no possible sarcopenia), and 1–3 quartiles were sequentially defined as severe, moderate, and mild possible sarcopenia. All-cause mortality data were retrieved in May 2020 from the National Civil Registration and Vital Statistics System, Ministry of Interior, Thailand.

Sociodemographic and clinical data, including age, gender, residential region, years of education, smoking status, and underlying diseases, were collected. Hypertension (HTN) was defined as systolic blood pressure (SBP) ≥140 mmHg or diastolic blood pressure (DBP) ≥90 mmHg or self-reported diagnosis of hypertension or use of any antihypertensive medications. Diabetes was defined as a fasting plasma glucose level of ≥7.0 mmol/L or self-reported diagnosis of diabetes or use of antidiabetic medications. Chronic kidney disease (CKD) was defined as an estimated glomerular filtration rate (eGFR) <60 mL/minute calculated using the Chronic Kidney Disease Epidemiology Collaboration equation (11). Depression was diagnosed using the criteria published in the Diagnostic and Statistical Manual of Mental Disorders, Fourth Edition (DSM IV) (12). Cerebrovascular disease (CVD) was defined by the self-reported diagnosis of CVD. Cardiovascular disease was also defined by the self-reported diagnosis of coronary heart diseases. Assessment of an individual's ability to perform activities of daily living (ADL) was measured using the Barthel Index (BI) (13). Impaired ADL was defined as the need for partial or total assistance in carrying out any basic ADL.

### Statistical Analysis

All the statistical analyses were performed using STATA software 16.1 (StataCorp LP, College Station, TX, USA). Study data were sample weighted against the total national registered population of Thailand in 2009, and methods for complex survey design analysis were applied (14). The baseline characteristics of study subjects were compared between groups using descriptive statistics. Parametric and non-parametric tests were applied depending on the distribution of data. Kruskal-Wallis test and chi-square test were used for continuous and categorical variables, respectively. A *p* < 0.05 was considered statistically significant for all tests. The association between groups and all-cause mortality was explored using univariate and multivariate Cox proportional hazards models. The proportional hazards assumption was checked on the basis of Schoenfeld residuals. There was no evidence that the proportional-hazards assumption was violated. Multivariate models were adjusted for potentially confounding factors, including age, residential area, diabetes, chronic kidney disease, cardiovascular disease, cerebrovascular disease, and history of smoking. The results of multivariate analysis are shown as hazard ratio (HR) and 95% confidence interval (CI) for univariate analysis, and as adjusted hazard ratio (aHR) and 95% CI for multivariate analysis.

### Ethical Considerations

Reporting of this study was in accordance with the Strengthening the Reporting of Observational Studies in Epidemiology (STROBE) guideline. The protocol for this study was approved by the Institutional Review Boards of both the Faculty of Medicine Ramathibodi Hospital, Mahidol University, and the Faculty of Medicine Siriraj Hospital, Mahidol University. Written informed consent was not obtained for this study because our data was retrospectively collected, but written informed consent was obtained when participants were originally invited to participate in the NHES-IV survey.

## Results

A total 8,195 participants were analyzed in this study. The mean age was 69.2 ± 6.8 years, and nearly half of participants were men. Almost 46% of total participants required some assistance in performing ADL. The prevalence of underweight, normal weight, overweight, and obesity was 14.8, 38.6, 16.7, and 29.9%, respectively. Female participants had a mean BMI higher than that of male participants (23.9 vs. 22.5 kg/m^2^, respectively). The prevalence of possible sarcopenia was 38.9%. Regarding the BMI and possible sarcopenia categories, 61.3% of underweight participants; 42.9% of normal weight participants; 35.2% of overweight participants and 26.8% of obese participants had possible sarcopenia. Among male participants, 64.3% of underweight participants, 46.2% of normal weight participants, 35.6% of overweight participants, and 28.3% of obese participants had possible sarcopenia. For female participants, the possible sarcopenia was identified at 57.6% for underweight participants; 38.7% for normal weight participants; 34.9% for overweight participants; and 25.8% for obese participants.

**Figure 2:**
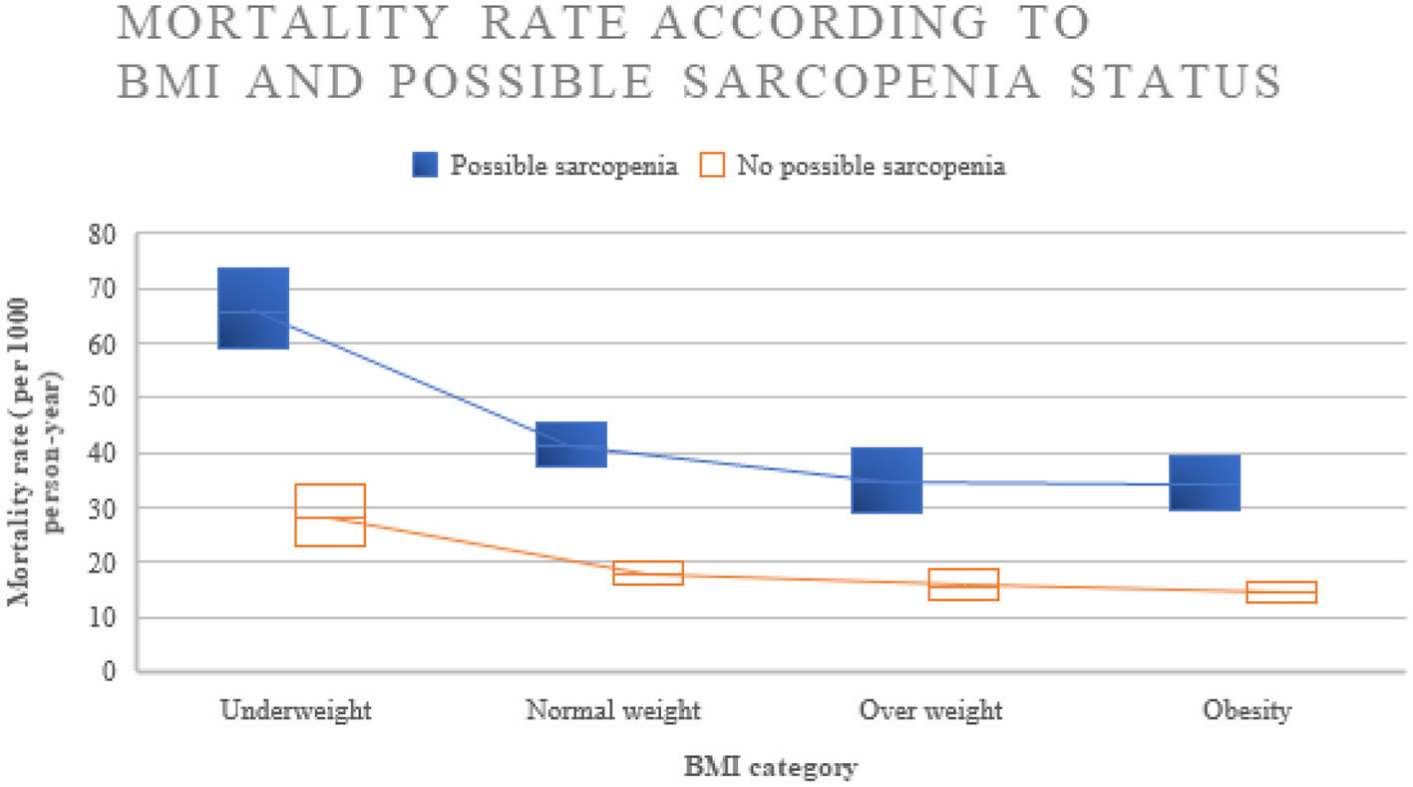
Mortality rate among older people with difference BMI and possible sarcopenia categories.

There were 1,771 deaths among our study population within 10 years of follow-up. Underweight and normal weight participants had a higher death rate compared to overweight and obese subjects. The all-cause mortality rate by BMI category (underweight, normal weight, overweight, and obesity) was 53.6 [95% confidence interval (CI): 47.2–60.8], 31.2 (95% CI: 28.4–34.2), 25.6 (95% CI: 21.8–30.0), and 25.0 (95%CI: 21.8–28.8) per 1,000 person-years, respectively, among older adult men, and 45.1 (95% CI: 38.8–52.4), 22.5 (95% CI: 20.0–25.5), 18.1(95% CI: 15.0–21.9), and 16.1 (95%CI: 14.1–18.4) per 1,000 person-years, respectively, among older adult women. When comparing according to BMI and possible sarcopenia status, participants with underweight with possible sarcopenia status had the highest mortality, while participants with obesity without possible sarcopenia status had the lowest mortality with death rates per 1,000 person-years of 65.7 (95% CI: 58.8–73.4) and 14.4 (95% CI: 12.7–16.4), respectively. The overall mortality according to BMI categories and possible sarcopenia status among included participants is shown in Figure 2. Sociodemographic, functional, lifestyle, clinical, mental health, and mortality characteristics of study participants for different BMI ranges compared between Thai older adults with and without possible sarcopenia are shown in Table 1.

**Table 1:** Sociodemographic, functional, lifestyle, clinical, mental health, and mortality characteristics of study participants for different body mass index (BMI) ranges compared between Thai older adults with and without possible sarcopenia (*N* = 8,195).

	**Total**	**BMI** **<** **18.5 kg/m**^**2**^ **(*****n*** **=** **1,090)**		**BMI 18.5–22.9 kg/m**^**2**^ **(*****n*** **=** **3,082)**		**BMI** ***2*****3.0–24.9 kg/m**^**2**^ **(*****n*** **=** **1,439)**		**BMI** **>** **25.0 kg/m**^**2**^ **(*****n*** **=** **2,584)**	
	**(*n* = 8,195)**	**With possible sarcopenia (*n* = 668, 61.3%)**	**Without possible sarcopenia (*n* = 422, 38.7%)**	**With possible sarcopenia (*n* = 1,321, 42.9%)**	**Without possible sarcopenia (*n* = 1,761, 57.1%)**	**With possible sarcopenia (*n* = 507, 35.2%)**	**Without possible sarcopenia (*n* = 932, 64.8%)**	**With possible sarcopenia (*n* = 692, 26.8%)**	**Without possible sarcopenia (*n* = 1,892, 73.2%)**
Age (year), mean (SD)	69.2 (6.8)	73.8 (7.2)	68.5 (6.3)	72.6 (7.1)	67.9 (6.2)	71.9 (7.2)	67.0 (5.5)	70.4 (6.8)	66-5 (5.4)
Male, *n* (%)	4,048 (49.4)	387 (57.9)	215 (51.0)	798 (60.4)	931 (52.9)	263 (51.9)	476 (51.1)	277 (40.0)	701 (37.1)
Education (year), mean (SD)	5.1 (3.1)	4.1 (1.8)	4.6 (2.5)	4.7 (2.6)	5.3 (3.3)	4.7 (2.9)	5.7 (3.6)	4.9 (2.9)	5.5 (3.5)
Impaired ADL, *n* (%)	3,736 (45.6)	353 (52.8)	174 (41.2)	646 (48.9)	683 (38.8)	262 (51.7)	346 (37.1)	389 (56.2)	883 (46.7)
Urban, *n* (%)	4,369 (53.3)	285 (38.6)	167 (39.6)	594 (45.0)	885 (50.3)	281 (55.4)	545 (58.5)	428 (61.9)	1,211 (64.0)
Smoking, *n* (%)	1,583 (19.3)	228 (34.1)	140 (33.2)	327 (24.8)	424 (24.1)	59 (11.6)	147 (15.8)	72 (10.4)	186 (9.8)
Hypertension, *n* (%)	4,098 (50.0)	224 (33.6)	120 (28.4)	625 (47.3)	721 (41.0)	292 (57.8)	485 (52.1)	458 (66.2)	1,173 (62.1)
Diabetes, *n* (%)	1,335 (16.3)	36 (5.4)	14 (3.3)	174 (13.2)	182 (10.3)	101 (19.9)	163 (17.5)	202 (29.2)	463 (24.5)
Chronic kidney disease, *n* (%)	2,368 (28.9)	184 (27.6)	82 (19.4)	459 (34.8)	403 (22.9)	184 (36.3)	255 (27.4)	239 (34.5)	562 (29.7)
Depression, *n* (%)	1,583 (19.3)	47 (7.0)	23 (5.5)	63 (4.8)	62 (3.5)	30 (3.9)	35 (3.8)	40 (5.8)	72 (3.8)
Cardiovascular disease, *n* (%)	347 (4.2)	21 (3.1)	10 (2.4)	56 (4.2)	43 (2.4)	25 (4.9)	25 (2.7)	37 (3.4)	57 (3.0)
Cerebrovascular disease, *n* (%)	251 (3.1)	31 (4.6)	6 (1.4)	39 (3.0)	31 (1.8)	29 (5.7)	33 (3.5)	54 (7.8)	101 (5.3)
Death per 1,000 person year (95% CI)	28.2 (27.0–29.4)	65.7 (58.8–73.4)	27.9 (22.8–34.1)	41.2 (37.5–45.4)	17.8 (15.8–20.1)	34.4 (29.2–40.7)	15.5 (13.0–18.5)	34.1 (29.6–39.3)	14.4 (12.7–16.4)

**Figure 3:**
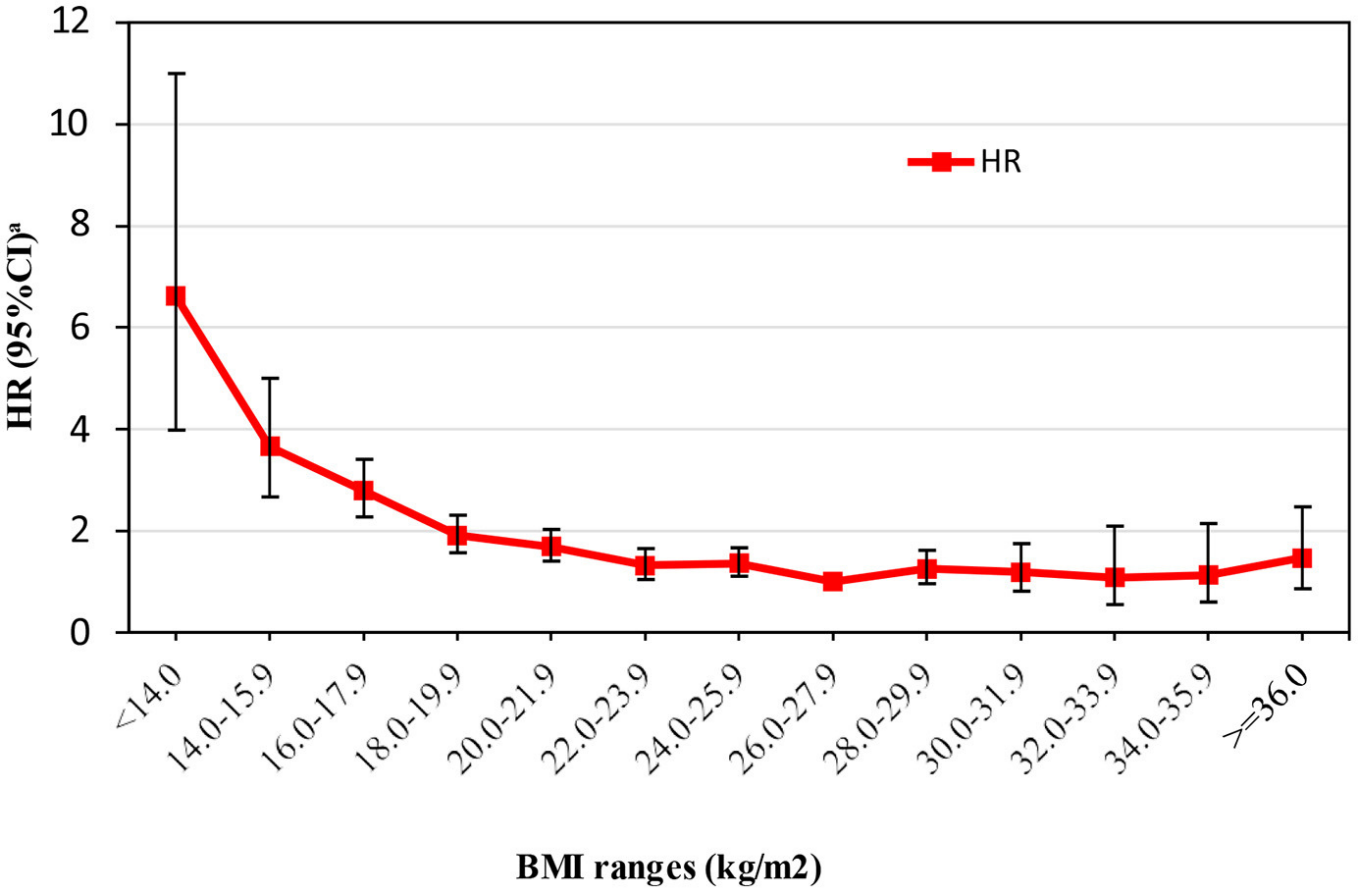
Association between the ranges of body mass index (BMI) and all-cause mortality after adjustment for potentially confounding factors. ^a^HR, hazard ratio; CI, confidence interval.

Association between the ranges of BMI and all-cause mortality after adjustment for potentially confounding factors is shown in Figure 3. BMI 26.0–27.9 kg/m^2^, the range with the lowest mortality, was defined as the reference group. Compared with the reference group, every range of BMI <26.0 kg/m^2^ was significantly associated with increased all-cause mortality, whereas the ranges of BMI > 28.0 kg/m^2^ were not significantly associated with increased mortality. Older adults with a BMI <14.0 kg/m^2^ had the highest risk for all-cause mortality with an adjusted hazard ratio (aHR) of 6.61 (95% CI: 3.98–10.99).

Association between 10-year all-cause mortality and BMI, possible sarcopenia status according to AWGS 2019, and possible sarcopenia severity according to quartile of handgrip strength among Thai older adults who participated in the NHES-IV is shown in Table 2. Concerning the BMI category, underweight status associated with a significant risk of mortality (aHR: 1.81, 95% CI: 1.58–2.07), whereas obesity was found to be a protective factor against death (aHR: 0.74, 95% CI: 0.63–0.87) when compared with the normal BMI group. Older adults in the overweight category appear to have similar mortality risk as those in the normal weight group. The results showed similar trends between genders.

**Table 2:** Association between 10-year all-cause mortality and body mass index (BMI), possible sarcopenia status according to AWGS 2019, and possible sarcopenia severity according to quartile of handgrip among Thai older adults who participated in the 4th Thai National Health Examination Survey (NHES-IV) (*N* = 8,195).

	**Total**		**Male**		**Female**	
	**Unadjusted**	Adjusted*	**Unadjusted**	Adjusted*	**Unadjusted**	Adjusted*
**BMI categories**				
Underweight (BMI <18.5)	1.93 (1.70–2.19)	1.81 (1.58–2.07)	1.99 (1.63–2.43)	1.87 (1.53–2.29)	1.92 (1.50–2.45)	1.85 (1.48–2.31)
Normal weight (BMI 18.5–22.9)	1.00 (Reference)	1.00 (Reference)	1.00 (Reference)	1.00 (Reference)	1.00 (Reference)	1.00 (Reference)
Overweight (BMI 23.0–24.9)	0.86 (0.74–1.00)	0.91 (0.78–1.05)	1.03 (0.87–1.22)	0.99 (0.82–1.18)	0.71 (0.56–0.89)	0.81 (0.64–1.03)
Obesity (BMI > 25.0)	0.70 (0.60–0.82)	0.74 (0.63–0.87)	0.83 (0.68–1.02)	0.71 (0.57–0.89)	0.68 (0.54–0.87)	0.83 (0.66–1.04)
**Possible sarcopenia categories**				
Possible sarcopenia	2.70 (2.45–2.98)	1.89 (1.70–2.09)	2.66 (2.27–3.12)	1.97 (1.65–2.35)	2.57 (2.20–3.00)	1.78 (1.51–2.11)
No possible sarcopenia	1.00 (Reference)	1.00 (Reference)	1.00 (Reference)	1.00 (Reference)	1.00 (Reference)	1.00 (Reference)
**Possible sarcopenia severity**				
Severe possible sarcopenia (6.5–24.6 kg in male, 6–16.6 kg in female)	4.11 (3.45–4.89)	2.46 (1.01–3.01)	4.26 (3.48–5.21)	2.92 (2.25–2.78)	3.95 (3.11–4.99)	2.21 (1.72–2.85)
Moderate possible sarcopenia (24.7–29.2 kg in male, 16.7–19.7 kg in female)	2.60 (2.25–3.00)	1.92 (1.65–2.23)	2.77 (2.25–3.41)	2.19 (1.75–2.74)	2.42 (1.90–3.08)	1.72 (1.35–2.20)
Mild possible sarcopenia (29.3–33.8 kg in male, 19.8–22.8 kg in female)	1.59 (1.37–1.84)	1.39 (1.20–1.61)	1.61 (1.24–2.08)	1.48 (1.14–1.91)	1.58 (1.27–1.96)	1.32 (1.05–1.67)
No possible sarcopenia (33.9–54.5 kg in male, 22.9–49.0 kg in female)	1.00 (Reference)	1.00 (Reference)	1.00 (Reference)	1.00 (Reference)	1.00 (Reference)	1.00 (Reference)

*Adjusted by age, residential area, diabetes, chronic kidney disease, cardiovascular disease, cerebrovascular disease, history of smoke.

Concerning possible sarcopenia status and defining older adults using the AWGS 2019 cut-off point, participants with possible sarcopenia had significantly higher mortality risk compared to those without possible sarcopenia (aHR: 1.89, 95% CI: 1.70–2.09). When classifying older adults by severity of possible sarcopenia according to quartile group of handgrip strength, the mortality risk was increased in a dose-response manner according to the severity of possible sarcopenia. Older adults with severe possible sarcopenia had the highest risk for mortality (aHR: 2.46, 95% CI: 1.01–3.01) compared to those with moderate or mild possible sarcopenia (aHR: 1.92, 95% CI: 1.65–2.23, and aHR: 1.39, 95% CI: 1.20–1.61, respectively). Similar results were observed between genders.

Association between 10-year all-cause mortality and BMI category combined with possible sarcopenia status for all study participants, and compared between male and female Thai older adults who participated in the NHES-IV is shown in Table 3. Multivariate analysis showed that older adults who were underweight with possible sarcopenia were at highest risk for mortality (aHR: 2.81, 95% CI: 2.32–3.40), whereas those who were obese without possible sarcopenia were at lowest risk for mortality (aHR: 0.67, 95% CI: 0.55–0.81) when using normal weight with no possible sarcopenia as the reference group. Our results showed improved classification for defining the risk of death among older adults when BMI and possible sarcopenia status were combined. Among normal weight and overweight older adults, having possible sarcopenia significantly increased mortality risk. In the underweight group, although possible sarcopenia and no possible sarcopenia both exerted increased risk of death, the possible sarcopenia group had substantially higher risk. In the obese group, having possible sarcopenia increased the risk of death with an aHR of 1.53 (95% CI: 1.25–1.88), whereas not having possible sarcopenia was a protective factor against death with an aHR of 0.67 (95% CI: 0.55–0.81). These associations were more pronounced in males compared to females.

**Table 3:** Association between 10-year all-cause mortality and body mass index (BMI) category stratified by possible sarcopenia status for all study participants, and compared between male and female Thai older adults who participated in the 4th Thai National Health Examination Survey (NHES-IV) (*N* = 8,195).

**BMI categories**	**Possible sarcopenia categories**	**Total participants**		**Male**		**Female**	
		**Unadjusted**	Adjusted*	**Unadjusted**	Adjusted*	**Unadjusted**	Adjusted*
Underweight (BMI <18.5)	With	4.00 (3.35–4.79)	2.81 (2.32–3.40)	4.07 (3.00–5.52)	3.05 (2.21–4.22)	3.84 (2.89–5.11)	2.66 (2.00–3.53)
	Without	1.53 (1.12–2.09)	1.58 (1.56–2.16)	1.46 (0.98–2.17)	1.54 (1.04–2.29)	1.62 (1.06–2.50)	1.65 (1.07–2.55)
Normal weight (BMI 18.5–22.9)	With	2.26 (1.87–2.74)	1.60 (1.31–1.95)	2.25 (1.77–2.84)	1.67 (1.30–2.15)	2.18 (1.62–2.94)	1.47 (1.07–2.01)
	Without	1.00 (Reference)	1.00 (Reference)	1.00 (Reference)	1.00 (Reference)	1.00 (Reference)	1.00 (Reference)
Overweight (BMI 23.0–24.9)	With	1.88 (1.53–2.32)	1.37 (1.10–1.72)	2.05 (1.61–2.60)	1.36 (1.03–1.79)	1.73 (1.25–2.39)	1.42 (1.04–1.95)
	Without	1.01 (0.74–1.40)	1.00 (0.71–1.40)	1.40 (0.95–2.06)	1.28 (0.85–1.92)	0.67 (0.47–0.94)	0.70 (0.48–1.03)
Obesity (BMI > 25.0)	With	2.16 (1.78–2.61)	1.53 (1.25–1.88)	3.10 (2.26–4.25)	1.77 (1.32–2.39)	1.70 (1.62–2.47)	1.43 (0.97–2.09)
	Without	0.70 (0.58–0.84)	0.67 (0.55–0.81)	0.68 (0.51–0.91)	0.54 (0.39–0.76)	0.73 (0.57–0.94)	0.81 (0.62–1.04)

*Adjusted by age, residential area, diabetes, chronic kidney disease, cardiovascular disease, cerebrovascular disease, history of smoke.

Association between 10-year all-cause mortality and BMI category combined with severity of possible sarcopenia for all study participants, and compared between male and female Thai older adults who participated in the 4th Thai NHES-IV is shown in Table 4. Multivariate analysis revealed older adults with underweight and severe possible sarcopenia status to be at the highest risk for increased mortality (aHR: 3.98, 95% CI: 2.89–5.48) when using normal weight without possible sarcopenia status as the reference group. In the overweight group, individuals with moderate-to-severe possible sarcopenia showed higher mortality compared to the reference group. Among those in the obesity group, having moderate-to-severe possible sarcopenia posed a higher risk of death, while the opposite was found among those who were obese without possible sarcopenia. Multivariate analysis showed the strongest risk of mortality among older adult males with underweight and severe possible sarcopenia status (aHR: 5.35, 95% CI: 1.19–8.97). In stark contrast, obesity without possible sarcopenia status was a protective factor against mortality (aHR: 0.61, 95% CI: 0.38–0.97).

**Table 4:** Association between 10-year all-cause mortality and body mass index (BMI) category stratified by possible sarcopenia severity for all study participants, and compared between male and female Thai older adults who participated in the 4th Thai National Health Examination Survey (NHES-IV) (*N* = 8,195).

**BMI categories**	**Possible sarcopenia severity**	**Total participants**		**Male**		**Female**	
		**Unadjusted**	Adjusted*	**Unadjusted**	Adjusted*	**Unadjusted**	Adjusted*
Underweight (BMI <18.5)	Severe	6.82 (4.92–9.47)	3.98 (2.89–5.48)	8.05 (5.00–12.98)	5.35 (1.19–8.97)	5.64 (3.76–8.48)	3.12 (2.06–4.72)
	Moderate	4.09 (2.94–5.67)	2.97 (2.13–4.14)	4.36 (2.95–6.47)	3.35 (2.27–4.95)	3.83 (2.34–6.26)	2.81 (1.75–4.53)
	Mild	2.83 (1.83–4.38)	2.72 (1.75–4.23)	3.03 (1.70–5.38)	3.08 (1.69–5.64)	2.70 (1.54–4.73)	2.56 (1.46–4.48)
	None	0.76 (0.40–1.47)	0.78 (0.41–1.51)	0.63 (0.26–1.54)	0.65 (0.27–1.58)	0.85 (0.39–1.89)	0.92 (0.43–2.01)
Normal weight (BMI 18.5–22.9)	Severe	3.73 (2.71–5.13)	2.18 (1.59–3.00)	4.23 (2.95–6.05)	2.74 (1.86–4.04)	3.22 (1.99–5.19)	1.79 (1.11–2.86)
	Moderate	2.79 (1.92–4.05)	1.99 (1.40–2.82)	3.20 (1.97–5.21)	2.37 (1.46–3.85)	2.31 (1.42–3.76)	1.64 (1.02–2.64)
	Mild	1.85 (1.33–2.57)	1.50 (1.10–2.03)	2.12 (1.46–3.04)	1.77 (1.21–2.59)	1.58 (0.91–2.73)	1.28 (0.75–2.18)
	None	1.00 (Reference)	1.00 (Reference)	1.00 (Reference)	1.00 (Reference)	1.00 (Reference)	1.00 (Reference)
Overweight (BMI 23.0–24.9)	Severe	3.45 (2.34–5.09)	2.05 (1.39–3.02)	4.36 (2.83–6.72)	2.45 (1.49–4.05)	2.85 (1.73–4.70)	1.89 (1.15–3.10)
	Moderate	2.21 (1.64–2.97)	1.58 (1.19–2.10)	3.13 (2.22–4.45)	2.13 (1.49–3.06)	1.44 (0.86–2.40)	1.13 (0.67–1.89)
	Mild	1.38 (0.91–2.12)	1.17 (0.77–1.78)	1.58 (0.82–3.02)	1.26 (0.66–2.40)	1.20 (0.67–2.15)	1.06 (0.59–1.89)
	None	1.63 (0.81–3.29)	1.48 (0.74–2.96)	2.85 (1.30–6.25)	2.44 (1.09–5.44)	0.67 (0.31–1.42)	0.65 (0.30–1.39)
Obesity (BMI > 25.0)	Severe	3.45 (2.45–4.85)	1.98 (1.41–2.79)	5.60 (3.67–8.54)	2.68 (1.70–4.24)	2.64 (1.65–4.25)	1.76 (1.10–2.81)
	Moderate	2.20 (1.56–3.11)	1.53 (1.10–2.79)	3.80 (2.20–6.58)	2.20 (1.35–3.58)	1.61 (1.08–2.42)	1.26 (0.83–1.90)
	Mild	1.23 (0.87–1.74)	1.01 (0.71–1.42)	1.85 (1.16–2.96)	1.26 (0.79–1.99)	0.99 (0.64–1.53)	0.91 (0.58–1.41)
	None	0.87 (0.59–1.29)	0.77 (0.52–1.14)	0.82 (0.53–1.26)	0.61 (0.38–0.97)	1.01 (0.51–1.62)	0.98 (0.55–1.73)

*Adjusted by age, residential area, diabetes, chronic kidney disease, cardiovascular disease, cerebrovascular disease, history of smoke.

## Discussion

The results of this study affirm the finding that being slightly obese leads to better chance of survival among older adults. It has been shown that higher BMI associated with lower risk of death among older people (15–17). However, the proposed optimal BMI range varies widely (18, 19). A previous meta-analysis that reported that older adults with a BMI of 27.0–27.9 kg/m^2^ had the lowest mortality did not include Asian population in the analysis (16). The present study proposes a BMI of 26.0–27.9 kg/m^2^ as the optimal BMI for Asian older adults since it was found to be associated with the lowest mortality after adjusting for potential confounders.

The present study showed the prevalence of underweight status among older adults to be substantially high in Thailand at approximately 15%. This finding is similar to those reported from several studies conducted in Thailand (20, 21), and in other countries in Asia (15, 17). However, these figures are in stark contrast to the 3% rates reported from the US (22) and Europe (23). Moreover, our study emphasizes the higher mortality risk associated with underweight status (when compared to obese status) among community-dwelling Thai older adults. This association is similar to those reported from other Asian countries (15, 17), but is different from the results of studies conducted in non-Asian older adult populations that found that the mortality risk associated with underweight and overweight status increased at similar magnitudes (16, 23, 24). A similar trend toward association between underweight status and increased mortality was found in a previous study of a large volume of pooled data from younger Asian population (25); however, the magnitude of increased mortality risk appears to be stronger among older adults. The possible explanations for synergistic mortality risk among older people with underweight status might stem from the accumulation of several poor prognostic factors, such as risk of infection (26), risk of falls (27), severe functional impairment, and other geriatric syndromes (28). Importantly, some of these factors are potentially modifiable. Since underweight status is one of those potentially modifiable factors, underweight status should be addressed at the healthcare policy level so that appropriate nutritional strategies can be developed and implemented, especially among Asian older adults.

In this study, we found the mortality rate of Thai older people was higher than the European countries (29). Thailand, a middle-income country, has been undergoing an epidemiological transition with predominant of non-communicable diseases. Cardiovascular diseases accounted for almost one-third of all deaths in Thai population (30). Compared to populations in America and Europe with different stages of economic development and earlier stage of cardiovascular epidemic; the mortality rate in Thai population as with other low-and middle-income countries are relatively higher than in high-income countries where cardiovascular and NCD death rates are lower (31). This might be due to the differences in socioeconomic, health system resources, and system performance for rate of awareness, coverage and treatment and control of the condition.

In contrast to BMI alone, this study showed that the association between BMI and all-cause mortality in older adults was modified by possible sarcopenic status, and was dependent upon the severity of possible sarcopenia. Among underweight older adults, the subgroup without possible sarcopenia was not at elevated risk for mortality; however, the risk of mortality increased in dose-response manner as the severity of possible sarcopenia increased. Furthermore, the classification of possible sarcopenia severity according to quartile of handgrip strength was also able to differentiate mortality risk among older adults in other weight groups. Among obese older adults, severity of possible sarcopenia improved the ability to assess and differentiate mortality risk. Obese people with moderate-to-severe possible sarcopenia were at increased risk, while those without possible sarcopenia realized protective effect relative to mortality risk.

Sarcopenia in older adults increases their mortality risk, as shown in a previous meta-analysis (32). The association between severity of sarcopenia using handgrip strength and all-cause mortality has also been addressed in previous studies (33, 34). The combination of BMI and sarcopenia, however, has been focused mostly on sarcopenia and obesity, namely sarcopenic obesity, as a condition of increased mortality risk (35). This finding has not been widely addressed in the literature. The mechanisms of sarcopenic obesity are based on changes in metabolism and body composition due to aging combined with poor physical activity from both physical and mental illnesses (36). The present study demonstrated similar association with mortality in the sarcopenic obesity group. However, the magnitude of risk in the present study was more pronounced in the possible sarcopenic-undernutrition group. A recent review by the UK group (37) included studies that investigated association between body composition and mortality, and they found no studies that used the combination of BMI and sarcopenia in other form apart from sarcopenic obesity. A previous study from Taiwan (38) reported synergistic mortality risk between the combination of low physical activity with underweight status and low physical activity with sarcopenia. An explanation for this finding was not proposed.

The obesity paradox, which is defined as the finding that people with a higher BMI are associated with a reduced risk of mortality, has been repeatedly established in studies conducted among older adults. Several attempts have been made to explore the underneath mechanism for protective effect of obesity paradox by combining additional measurements. A previous systematic review (39) explored the effect of physical fitness on this phenomenon in older adults, but they failed to arrive at a robust conclusion, and they commented on the limited validity of physical activity measurement in the included studies. A recent study (40) attempted to stratify risk among middle-aged adults according to BMI using cardiorespiratory fitness. They reported that fitness modified mortality risk in obese men, but not in obese women. The present study proposes the explanation for obesity paradox by further stratified BMI using possible sarcopenia status. We discovered a similar trend to previous study using sarcopenic status as a fitness measurement for older adults. The combination of BMI and possible sarcopenic status could better classify obese older adults into different risk groups, which is in accordance with the “fat, but fit” phenomenon (39).

Possible sarcopenic status in combination with BMI appears to be a good body composition measurement for classifying mortality risk among older adults. Possible sarcopenia enhances mortality risk after classified by BMI in all categories. Previous studies attempted to classify risk groups for mortality using several body composition parameters that ranged from a single parameter to a combination of parameters; however, their results were inconclusive (37). One explanation could be that the relationships between these parameters and mortality are not linear and the method of combining matters.

The main strengths of this study include the use of data from a nationwide survey from a representative group of population with a long-term follow-up period. The mortality outcome was assessed using mortality data from a national registry that has been affirmed as a reliable database for mortality data in Thailand. Moreover, the BMI categories used ethnicity-specific cut-off points that were developed for Asia-Pacific population. This study also used a recent definition of possible sarcopenia that is specific for Asian population. The classification of possible sarcopenia severity using quartile of handgrip strength demonstrated robustness by showing a similar trend in all subgroups, and the relationship with mortality risk was in a dose-response manner. The important limitation is that all information was collected only once during 2008–2009, it is unknown whether those anthropometric measures changed during last years of life. Additionally, many confounding factors derived from self-report information, such as history of stroke or cardiovascular disease, might be vulnerable to underreporting bias. Furthermore, similar to other cohort study, residual confounding factors could also be an issue in the present study.

### Implications for Policy and Practice

Optimal nutritional status is considered to be a key component of healthy aging. This study demonstrates that a combination of anthropometric measurements may be a better tool, compared to BMI alone, for guiding optimal nutritional status, and for reducing mortality risk. Moreover, parameters need to be age group and ethnicity specific to improve the accuracy and reliability of risk assessment. Among older adults, an attempt should be made to maintain a slightly higher BMI, and to avoid sarcopenic status. In addition to a proper diet, resistance exercise is approved for improving muscle strength, muscle mass, and physical function (41, 42). Therefore, an exercise program should be included in the health promoting package in all settings (43). Among older adults who are underweight, recommendations should be made to encourage increased both body weight and muscle strength. This would include adequate total caloric intake and increased protein intake in combination with resistance exercise. Among those with overweight/obesity status, possible sarcopenic status should be measured. Appropriate recommendations should then be given. Optimal resistance exercise should be considered in all older people with possible sarcopenia.

In conclusion, the results of this study revealed that the combination of BMI and possible sarcopenia could be a better predictor of all-cause mortality among Asian older adults compared to either parameter alone. In addition to BMI measurement, handgrip strength should be included as a nutritional assessment tool. Multivariate analysis showed underweight individuals with severe possible sarcopenia to be at highest risk for increased mortality, and higher risk was found in men. Obese status without possible sarcopenia was found to be an independent protective factor against mortality. Among older adults with a high BMI, proper diet and resistance exercise should be encouraged to prevent sarcopenia as opposed to encouraging weight reduction alone. “Fat, but fit” might be the most favorable outlook for older people.

## Data Availability Statement

The original contributions presented in the study are included in the article. Further inquiries can be directed to the corresponding author.

## Ethics Statement

The studies involving human participants were reviewed and approved by Human Research protection Unit, Faculty of Medicine Siriraj Hospital, Mahidol University. The patients/participants provided their written informed consent to participate in this study.

## Author Contributions

CC, VS, and WA: conceptualization, formal analysis, and review and editing of the manuscript. WA: methodology, resources, data curation, and project administration. CC: software. CC and VS: writing—original draft of the manuscript. VS: supervision. All authors have read and are in agreement with the submitted version of the manuscript.

## Funding

This study was supported by a grant from National Research Council of Thailand grant number 127/2564, the Faculty of Medicine Siriraj Hospital grant number: R015932039, and Mahidol University, Bangkok, Thailand.

## Conflict of Interest

The authors declare that the research was conducted in the absence of any commercial or financial relationships that could be construed as a potential conflict of interest.

## Publisher's Note

All claims expressed in this article are solely those of the authors and do not necessarily represent those of their affiliated organizations, or those of the publisher, the editors and the reviewers. Any product that may be evaluated in this article, or claim that may be made by its manufacturer, is not guaranteed or endorsed by the publisher.
